# Microstructure, Mechanical and Tribological Properties of Cold Sprayed Fe-Based Metallic Glass Coatings

**DOI:** 10.3390/ma18214875

**Published:** 2025-10-24

**Authors:** Anna Góral, Anna Trelka-Druzic, Wojciech Żórawski, Łukasz Maj, Martin Vicen, Otakar Bokůvka, Paweł Petrzak, Grzegorz Garzeł

**Affiliations:** 1 Institute of Metallurgy and Materials Science, Polish Academy of Sciences, 25 Reymonta St., 30-059 Kraków, Poland; a.trelka@imim.pl (A.T.-D.); l.maj@imim.pl (Ł.M.); p.petrzak@imim.pl (P.P.); g.garzel@imim.pl (G.G.); 2 Faculty of Mechatronics and Mechanical Engineering, Kielce University of Technology, Tysiąclecia Państwa Polskiego 7, 25-314 Kielce, Poland; ktrwz@tu.kielce.pl; 3 Department of Materials Engineering, Faculty of Mechanical Engineering, University of Žilina, 010 26 Žilina, Slovakia; martin.vicen@fstroj.uniza.sk (M.V.); otakar.bokuvka@fstroj.uniza.sk (O.B.)

**Keywords:** amorphous coating, cold spraying, microstructure, microhardness, flexural strength, wear resistance

## Abstract

Iron-based metallic glasses are gaining increased interest due to their good glass-forming ability, high compressive strength, high corrosion resistance, catalytic properties, excellent soft magnetic properties, and relatively low cost. Cold spraying was successfully used to produce amorphous coatings from commercially available powder without any crystallization due to its high cooling rate and short processing time, minimizing thermal influences. Thick and dense amorphous coatings were obtained. The effect of a substrate on the microstructure, phase composition, microhardness, flexural strength, and wear behaviour of the coatings was investigated. The cold sprayed coatings revealed an almost complete amorphous structure and negligible porosity. The coating deposited on the steel substrate showed higher microhardness, better resistance to loose abrasive wear, and a slightly lower wear index tested in the coating and Si_3_N_4_ ball tribological association than that cold sprayed on an Al alloy. The force required to destroy the durability of the coating–steel substrate system estimated during three-point bending tests was also much higher. Both coatings were characterized by a comparable friction coefficient.

## 1. Introduction

Metallic glasses (MGs) have unique properties resulting from their disordered atomic structure, lack of grain boundaries, and crystal lattice defects. Unusual properties such as specific strength, elastic limit, high hardness, exceptional corrosion and wear resistance, and unique magnetic properties make these materials attractive for many applications [1,2,3,4,5,6,7,8]. However, bulk metallic glasses have limited plasticity at room temperature, which limits their commercial use. The small critical size caused by poor glass formability and internal brittleness significantly hinders their extensive use as utility materials [9,10]. These materials are used in the coating form to overcome the size limitation and expand their practical application. Their unique properties make metallic glasses an interesting material in surface engineering, and they are used to protect different elements against wear and corrosion. A coating is formed from small metallic glass particles due to their increased plasticity at temperatures above the glass transition temperature [11,12].

Various thermal spray processes (e.g., arc, flame, high velocity oxygen fuel (HVOF), plasma, cold gas) are used to create amorphous coatings [13,14]. In each of these processes, two factors play a fundamental role: a heat source responsible for increasing the temperature of the sprayed material and a gas that accelerates and deposits the sprayed material particles. These processes provide possibilities for shaping the properties of amorphous sprayed coatings. However, in most of the mentioned processes, high temperatures present during spraying can lead to excessive oxidation, causing partial crystallization and affecting the phase composition, microstructure and properties of the coating [15,16,17,18]. The possibility of eliminating these unfavourable processes can be provided by the cold spraying technique. In this process, particles of the metal powder are deposited at the moment of impact on the substrate, resulting from their severe plastic deformation occurring below the melting temperature of the metal. In this way, during the spraying of the coating, the adverse effect of heat on the coating material and harmful processes related to particle oxidation and other problems occurring during conventional thermal spraying processes are eliminated [19,20,21,22]. The deposition mechanisms of amorphous coatings differ from those of crystalline metal coatings [23]. Understanding the mechanisms occurring during the plastic deformation of amorphous particles is key, because MGs are non-crystalline materials with short- or medium-range atomic configurations. During the cold spray process, amorphous powder particles hit the substrate at high speeds (300–1200 m/s) at a temperature below the powder melting point, forming a coating resulting from their intensive plastic deformation. A MG coating is formed by the deposition of successive layers formed by particle impact [24]. However, the mechanical properties and the thermodynamics of metallic glasses should be considered to ensure effective particle deposition. These materials crystallize above the temperature of crystallization, and their mechanical properties depend on the temperature and strain rate [25,26]. At temperatures well below the glass transition temperature (Tg), the particles are brittle, i.e., they do not undergo plastic deformation, which results in poor bonding with the substrate and coating cohesion. At high strain rates and temperatures slightly below Tg, the flow of the material is localised in thin shear bands, and the rest of the material remains undeformed, which means that there is so-called non-uniform flow [11,27,28,29]. At temperatures above Tg and low rate of strain, the homogeneous flow of the material takes place, and uniform deformation occurs for a sample subjected to uniform stress. The mechanisms that will be more pronounced depend on a specific material and its properties. A cold spray process is influenced by the powder’s phase composition, particle size distribution, grain morphology and thermal properties. Knowledge of the behaviour of amorphous powders during impact is fundamental to selecting the process parameters (gas type, pressure and temperature of the gas, spraying distance, and substrate hardness) at which MGs can be deposited during cold spraying. The phase composition depends on the cooling rate, which controls the amorphous phase stability and the ability to form glass [30]. The MG deposition mechanisms result from the dynamic conditions during impact and the rheological behaviour during cold spraying [26].

Fe-based metallic glasses have been intensively investigated due to their good glass-forming ability, high compressive strength, high corrosion resistance, good magnetic and catalytic properties, and relatively low cost [4,24,31,32,33]. Amorphous Fe-based alloys can also be promising candidates as thermal barrier coatings (TBC) on large tank inner hulls due to low thermal conductivity and crack resistance [13,34,35]. In recent years, the possibilities of producing amorphous Fe-based coatings with different chemical compositions by thermal arc spraying [36], detonation [37], plasma [14,38,39,40], supersonic [18,41], and cold spraying [18,24,28,40,42,43] have been tested, and their tribological and corrosion properties have mainly been analysed [13,18,37,38,39,40,41,44]. Amorphous coatings deposited by traditional thermal spray methods undergo partial crystallization and oxidation due to the high process temperature, which reduces their anti-corrosion and mechanical properties. The low temperature of the cold spray process enables the production of fully amorphous coatings with a compact structure and negligible porosity without signs of oxidation. Hence, cold spraying can be utilized to produce high-quality Fe-based amorphous coatings. As shown by Cao et al. [40] and Su et al. [18], coatings with a metallic glass structure sprayed with cold gas from Fe-based powders (Fe25Cr20Mo1Si and Fe48Cr15Mo14C15B6Y2, respectively) show better anti-corrosion and tribological properties than those sprayed with plasma. This is caused by the significantly lower porosity and high hardness of coatings sprayed with cold gas.

Hence, to fully utilise the potential of metallic glasses, further work is necessary to obtain thick amorphous coatings with suitable properties. The novelty of this work is aimed at creating thick, compact metallic glass coatings with good mechanical and tribological properties using the cold spray process. The thermal sprayed coatings were typically 100–450 µm thick [14,36,37,40], although the cold sprayed deposits can reach a thickness of up to 800–900 µm [18,42] after optimising the process parameters. There is some information in the literature on the formation mechanism of fully or partially amorphous various Fe-based coatings during cold spraying [24,26,28,42]; however, the characterization of mechanical and tribological properties is limited [18,40]. Su et al. [18] investigated Fe48Cr15Mo14C15B6Y2 coatings on steel 35CrMo and determined their microhardness, friction coefficient and wear rate. In turn, Cao et al. [40] showed only the hardness and weight loss of the Fe25Cr20Mo1Si coatings cold sprayed on the 40Cr steel. This study shows relationships between structures, hardness, flexural strength, and wear characteristics of coating-substrate systems as a function of the substrate used, either Al 7075 alloy or X6CrNiTi18-10 stainless steel. As shown in the literature [24,28], the mechanical and thermal properties of the substrate material significantly affect the deformation of metallic glass particles. They also influence the bonding and microstructure of metallic glass coatings made by cold spraying. Further studies on coating–substrate system behaviour during three-point bending and tribological tests will help clarify their use in abrasive wear conditions.

## 2. Materials and Methods

Kuamet 6B2 powder with chemical composition (in wt.%) of 87.41 Fe, 6.74 Si, 2.57 B, 2.53 Cr, and 0.75 C (Epson Atmix Corporation, Aomori, Japan) was chosen as the feedstock. In the paper, it is referred to as FeSiBCrC. The powder was cold sprayed both on Al 7075 alloy and X6CrNiTi18-10 steel substrates in the form of flat bars with dimensions of 400 mm × 30 mm × 5 mm grit-blasted with F30 electro-corundum (600–710 µm). The chemical compositions of the substrates are given in Table 1. The substrates were differentiated by hardness, which for the steel substrate was 220 HV1 and for the Al alloy was 177 HV1. The cold spray process was performed using an Impact Innovations 5/8 (Impact Innovations GmbH, Rattenkirchen, Germany) cold spray system. The process parameters are presented in Table 2.

The morphology and cross-sections of the powder and the surface morphology and cross-sections of cold sprayed coatings were investigated using a scanning electron microscope (FEI/Philips XL30, FEI Company, Hillsboro, OR, USA). To examine the phase structure, a Bruker D8 Discover diffractometer (Bruker AXS GmbH, Karlsruhe, Germany) (CoKα radiation) was used. The phase analysis was performed using Diffrac. EVA 3.0 and HighScore Plus 4.8 software with the PDF-5 database. The coating porosity was measured based on the coating cross-section microstructures (10 images) using the binary segmentation method in ImageJ 1.52a software. The thin foils for TEM observations were cut with the Focused Ion Beam (FIB) technique using a ThermoFisher Scios 2 Dual Beam microscope (Thermo Fisher Scientific, Waltham, MA, USA). A ThermoFisher Themis G2 200 kV FEG transmission electron microscope (Thermo Fisher Scientific, Waltham, MA, USA) equipped with a ChemiSTEM™ energy dispersive X-ray spectrometer (EDS), was used for the coating structure observations, especially near the interface zone. Calorimetric studies of powder and coatings cut from substrates were carried out using a Netzsch DSC F1 404 differential scanning calorimeter (NETZSCH-Gerätebau GmbH, Selb, Germany) in an inert gas atmosphere of argon with 70 mL/min flow. The measurement range was from 20 °C to 880 °C with a rate of 10 °C/min. The Vickers microhardness (HV0.3) of the coatings was measured on their cross-sections using a Buehler Micromet 5103 tester (Buehler, Lake Bluff, IL, USA) and an average of eight measurements was used. The flexural strength of the obtained coating–substrate systems was determined using an INSTRON 6025 modernized by Zwick/Roell (Ulm, Germany) with a computer-controlled mandrel traverse speed. The three-point bending test was repeated three times for such systems. The test samples had the same coating and substrate thickness, and their dimensions were 1.2 mm × 3 mm × 24 mm. The test was performed under a constant speed of 0.001 mm/s of the counter-sample into the substrate–coating system at room temperature. The applied force increased, causing the coating to crack. The wear tests with loose abrasive were performed using a T-07 tester (dry sand-rubber wheel) manufactured by the Institute for Sustainable Technologies in Radom, Poland. The test consisted of rubbing the coating with a counter-sample in the form of a rubber wheel (Ø50 × 20 mm) at a rotational speed of 200 rpm, and simultaneously feeding loose abrasive material, in the form of Al_2_O_3_ particles in a size range from 250 μm to 300 μm, directly into the friction zone. The feeding rate of the abrasives was 250 g/min. The test was performed at a load of 50 N. The loss of mass of the coatings was analysed after the next 10 min of the process. The dry coefficient of friction (CoF) of the coatings and substrates was measured using a linear sliding tribometer with the ball as the counter sample. The method of preparing all samples for testing was the same and included grinding on sandpaper and then polishing on diamond pastes. During the test, 21,000 cycles of reciprocating motion of the Si_3_N_4_ ball (3 mm diameter) over the coating surface at the sliding speed of 0.1 m/s were performed. The test duration was 10,500 s (175 min). The test ball was loaded using the normal force of 5 N. All results of wear and CoF were taken from an average of three such tests. Measurements of the samples’ surface profile after wear tests were performed using a ProFilm 3D non-contact interferometric profilometer (Filmetrics, Inc., a KLA Company, Milpitas, CA, USA).

## 3. Results and Discussion

### 3.1. Characterization of the Powder

**Figure 1:**
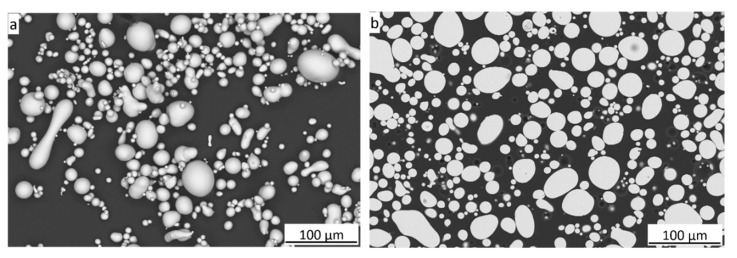
SEM morphologies (**a**) and cross-sections (**b**) of the FeSiBCrC feedstock.

**Figure 2:**
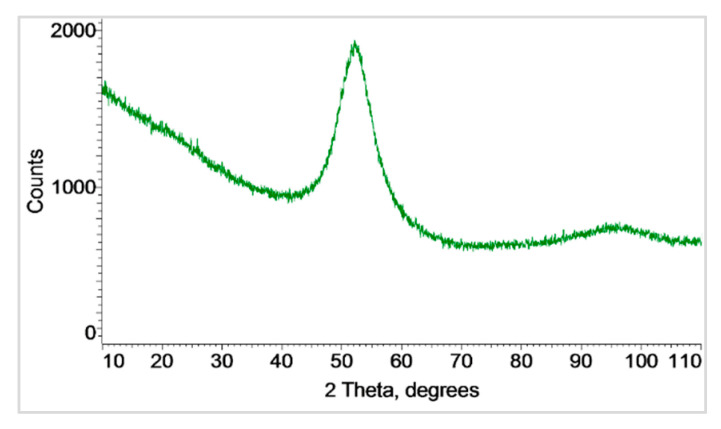
XRD pattern of the amorphous FeSiBCrC powder.

The SEM morphologies and cross-sections of the feedstock powder are shown in Figure 1. Its spherical shape is beneficial to the particle deformation and bonding. The powder has a smooth surface. The grain size given by the manufacturer was d_10_ = 11 µm, d_50_ = 26.7 µm, and d_90_ = 49.5 µm. The powder possesses an almost amorphous structure, as confirmed by the X-ray diffraction (XRD) pattern, Figure 2. The determined amorphous phase was 93.0% in the powder.

**Figure 3:**
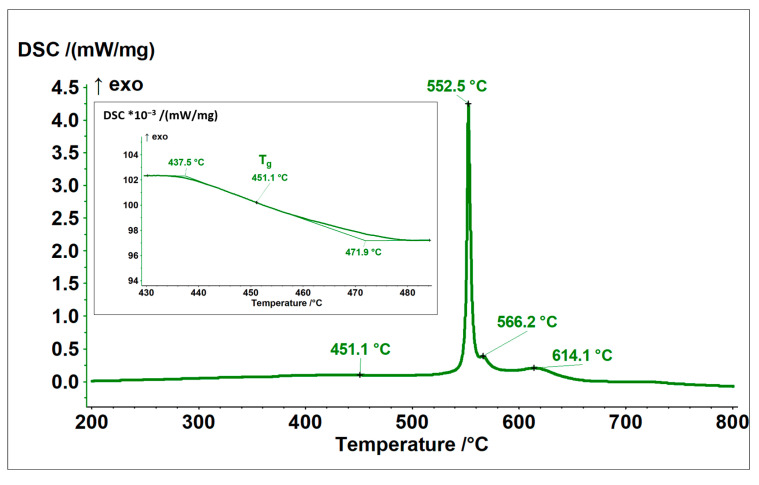
Thermal analysis of the FeSiBCrC powder.

Figure 3 shows DSC curves made for the powder. At a temperature of about 300 °C, the relaxation of the powder’s amorphous structure begins. The glass transition temperature was about 451.1 °C. The DSC curve made for the powder shows a high exothermic peak at 552.5 °C, which indicates the primary crystallization temperature, i.e., the transition from the amorphous state to the nanocrystalline or microcrystalline state. Two more exothermic peaks are observed at 566.2 °C and 614.1 °C, which indicate secondary crystallization and crystallite growth.

### 3.2. Characterization of the FeSiBCrC Cold Sprayed Coatings

#### 3.2.1. Coating Microstructure

**Figure 4:**
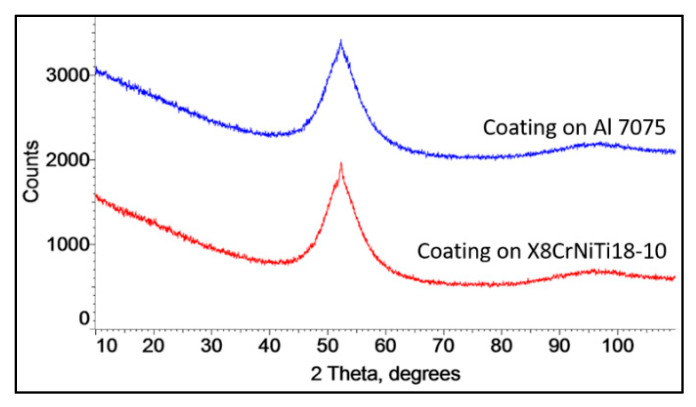
XRD patterns of the cold sprayed FeSiBCrC deposits.

The XRD investigations showed the amorphous structure of the coatings independently of the substrate. The XRD patterns for both coatings are the same. They show a broad halo peak at approximately 2θ = 45–60°, a characteristic of the amorphous phase (Figure 4). There was no evidence of phase crystallization during cold spraying, and an amorphous particle structure was mainly preserved. The estimated amorphous phase was 92.4% in the coating on the Al 7075 alloy and 92.2% in the coating on the steel substrate.

**Figure 5:**
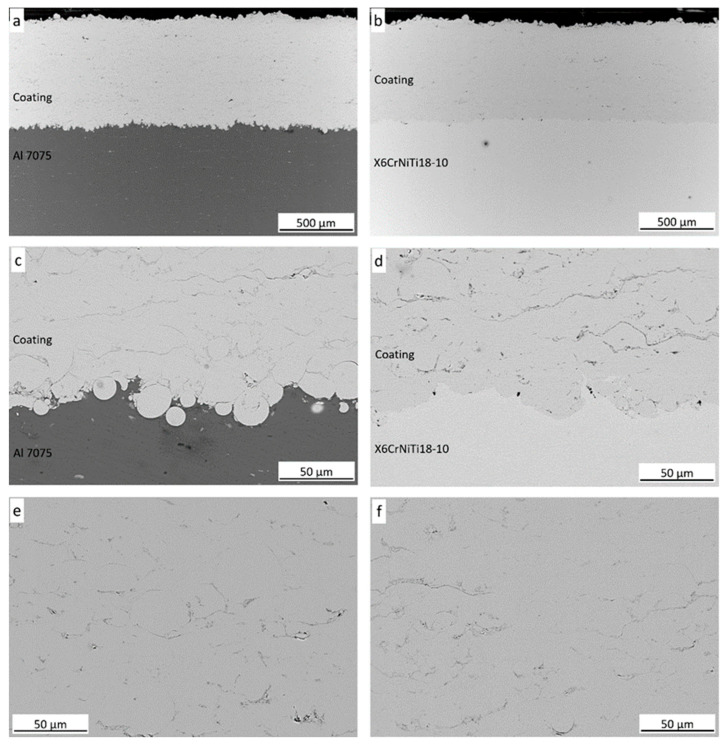
SEM-BSE (backscattered electron mode) microstructure of the deposits’ cross-sections on Al alloy (**a**,**c**,**e**) and steel (**b**,**d**,**f**).

Both coatings revealed compact and homogeneous microstructures with low porosity, not exceeding 2.5 vol.%, regardless of the substrate used (2.5 ± 0.3 vol.% for the coating sprayed on the Al alloy and 2.3 ± 0.4 vol.% for the one deposited on steel). The porosity observed was similar to that found in other cold sprayed coatings; for example, 2.0% [18], 2.3% [28], and 2.97% [40]. The particles appear to be tightly bonded and form dense coatings (Figure 5). The pores were observed mainly between deformed particles. The absence of discontinuities in the deposit–substrate interface zones confirmed the high coating adhesion. The cold sprayed coatings on the Al alloy had a thickness of 740 ± 34 µm and 703 ± 25 µm when deposited on steel. This was slightly lower than the Fe48Cr15Mo14C15B6Y2 deposited on 35CrMo steel (900 μm) [18], comparable to the Kuamet coatings cold sprayed by Henao et al. (800 μm) [42] and the Fe44Co6Cr15Mo14C15B6 deposited by List et al. (600–750 μm) [28], as well as about three times higher than the Fe25Cr20Mo1Si deposited on a 40Cr (230–270 μm) [40]. FeSiBCrC metallic glass coatings produced using cold spraying were thicker and had lower porosity than amorphous Fe-based coatings produced by other thermal spraying methods [14,36,37,41].

The coating cross-section shows mostly deformed particles. The particles have different diameters, and therefore have different velocities and experience different strengths during subsequent impact processes, leading to different deformation ratios. Moreover, the deposition mechanisms of metallic glasses in the cold spray process differ significantly from crystalline materials because of a long-range ordered structure, their thermal behaviour, and complex deformation mechanisms, i.e., the lack of strain hardening and defects, such as dislocations, which are associated with plastic deformation of crystalline materials [29]. It has been shown that above Tg and below the crystallization temperature, particles impacting the substrate exhibit the highest deposition efficiency and the lowest coating porosity. This results in uniform deformation, which assists particle binding [26,28]. In contrast, below Tg, localized plastic flow occurs at the particle boundary and shear bands form. Shear bands show the localization of shear deformation under the influence of applied stress, which occurs by local rearrangements of atoms around free-volume regions [48,49]. The shear bands are thin (20 to 100 nm) and form rapidly after reaching the stress limit. The local temperature increase at the shear instability at the particle–particle interface can exceed Tg, which promotes further plastic deformation and particle bonding. The particles can flow more easily at impact. A condition for coating formation is that the softening due to shear instability at the particle surface occurs rapidly enough to reduce the loading inside the particle, thereby avoiding brittle or adiabatic shearing [28]. However, even at temperatures close to Tg, the particle deformation can be non-uniform at the strain rates occurring in the cold spray process.

The nature of the first layer depositing in the area near the substrate is also different. The first particles hitting the grit-blasted substrate penetrated the soft Al 7075 alloy almost without deformation. Particles deposited on the steel substrate are more deformed and do not penetrate deeply into the substrate. This effect is directly related to the hardness of the substrate materials. The X6CrNiTi18-10 steel had 25% higher hardness (220 HV1) than the Al 7075 alloy (177 HV1). High deformation rates and high particle deformation were observed on the hard substrate. The soft substrates absorb most of the kinetic energy during impact, leading to weak particle deformation. The more compact metallic glass coating is formed on the hard substrates. The Al 7075 alloy substrate shows lower strain hardening and strength and deforms more easily under similar stresses in contrast to the steel substrate [50]. The metal substrate’s thermal properties also influence the heat dissipation rate from the metallic glass particle during impact and its deformation [24]. Substrates with a higher diffusion coefficient cool the first layer of metallic glass faster.

**Figure 6:**
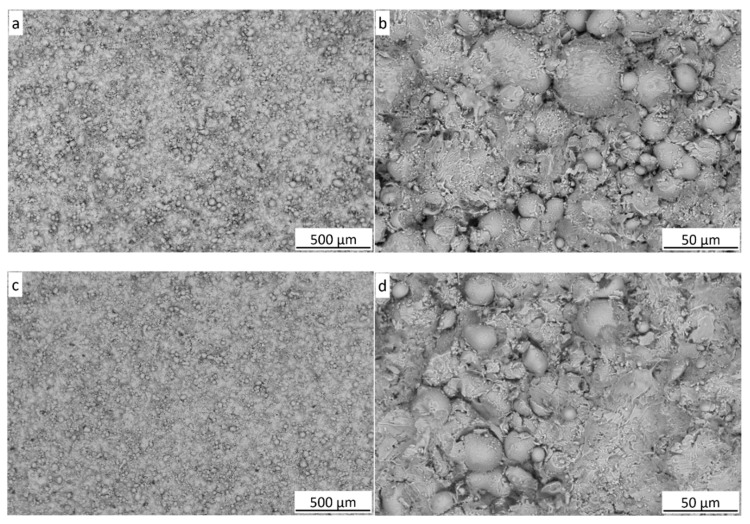
SEM-BSE surface morphology of the deposits on Al alloy (**a**,**b**) and steel (**c**,**d**).

The surface of the coatings, regardless of the substrate used, was characterized by strongly deformed amorphous phase particles, between which there were partially deformed and undeformed particles (Figure 6).

**Figure 7:**
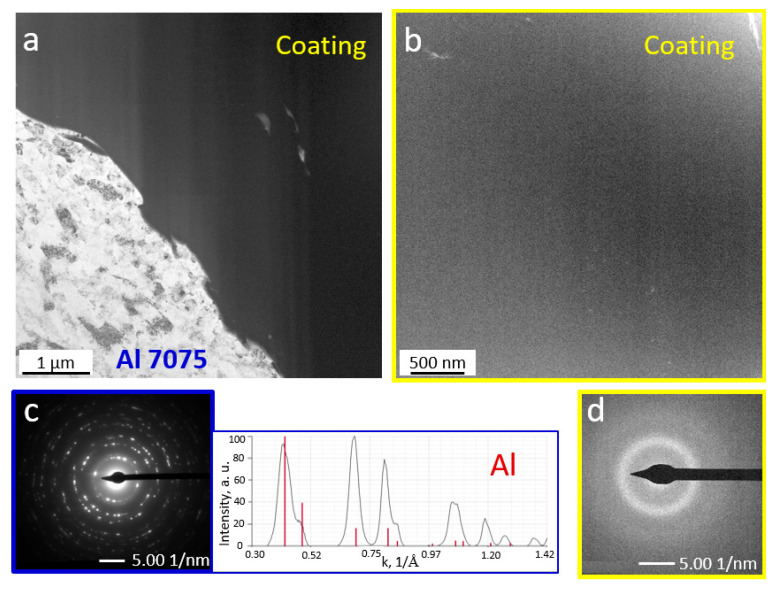
Bright field TEM images of coatings sprayed on the Al 7075 alloy substrate (**a**) and coating (**b**) with selected area diffraction patterns of the substrate (**c**) and coating (**d**).

**Figure 8:**
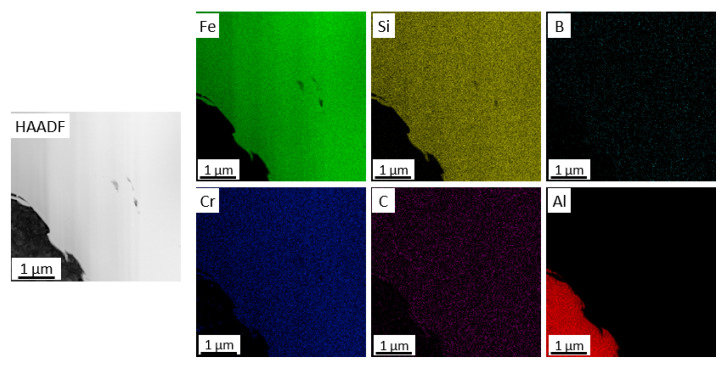
STEM image of the coating cold sprayed on Al 7075 alloy in the interface area and corresponding chemical element distribution maps.

**Figure 9:**
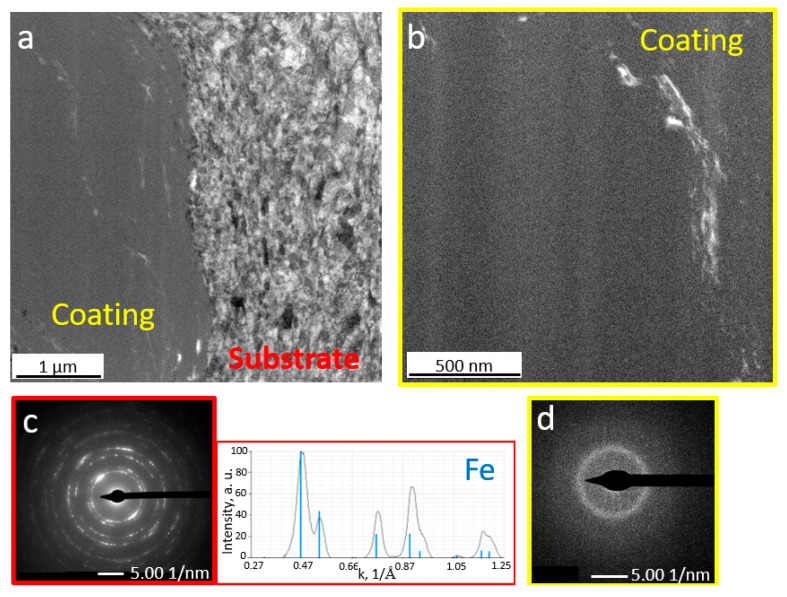
Bright field TEM images of coatings sprayed on the steel substrate (**a**) and coating (**b**) with selected area diffraction patterns of the substrate (**c**) and coating (**d**).

**Figure 10:**
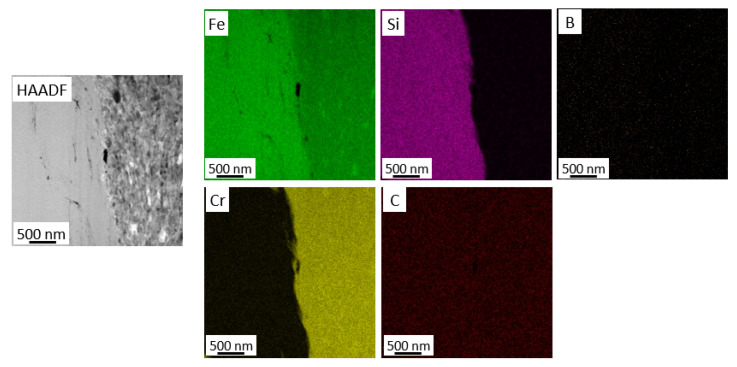
STEM image of the coating cold sprayed on the steel in the interface area and corresponding chemical element distribution maps.

Micro/nanoscale studies of the coatings allowed for characterization of the zone at the coating–substrate boundary (Figure 7, Figure 8, Figure 9 and Figure 10). Studies performed using TEM confirm more intensive deformation of the X6CrNiTi18-10 steel substrate (Figure 9) compared to the Al 7075 substrate (Figure 7), which is directly related to their properties, including hardness. Both coatings retained the amorphous feedstock structure. As the maps of chemical element distributions (Figure 8 and Figure 10) showed, the arrangement of all elements in the coating microstructure was uniform, independent of the substrate type.

#### 3.2.2. Mechanical and Tribological Properties of the Coatings

**Figure 11:**
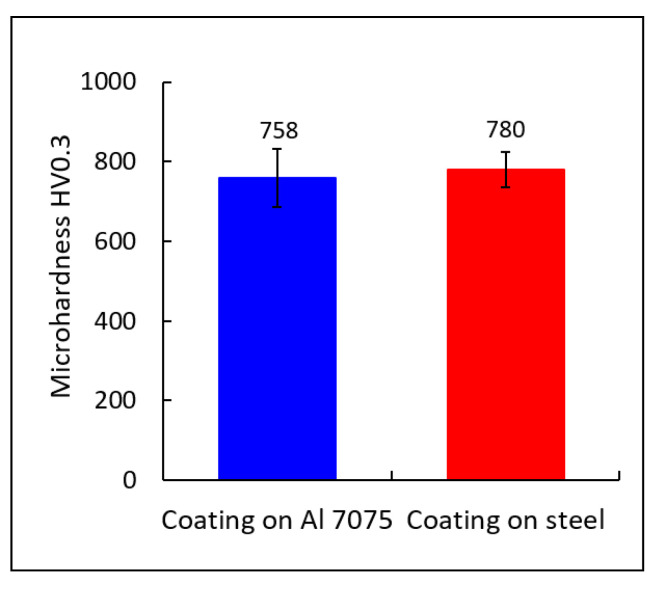
Microhardness of the coatings.

Coatings cold sprayed on the steel substrate had a slightly higher hardness (780 ± 44 HV0.3) than those sprayed on the Al alloy (758 ± 73 HV0.3), Figure 11. These values were approximately 3.5 and 4 times higher respectively than the hardness of the substrate materials (220 HV1 of the steel and 177 HV1 of the Al alloy) on which they were sprayed. A similar phenomenon was observed by Żórawski et al. [51] for cold sprayed Zr-based metallic glass deposits.

**Figure 12:**
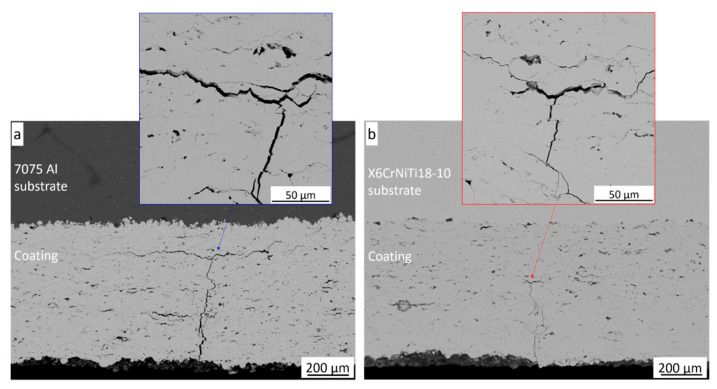
SEM-BSE cross-section microstructures of the investigated systems after the three-point bending test; the coating on al 7075 alloy (**a**), the coating on the steel (**b**).

The behaviour of the amorphous coatings cold sprayed on different substrates during the three-point bending test and their cracking susceptibility were investigated. The force was applied to the substrate side of the examined substrate–coating systems. The counter-sample moved at a constant speed into the substrate, causing the pressure force to increase until a crack occurred. In both cases, the crack appeared where the force was applied and ran from the surface into the coating. No peeling off of the coating occurred. The courses of the cracks are shown in Figure 12. With increasing force, a crack propagated across the deposit perpendicular to the surface. The crack paths ran mainly along the boundaries of the deformed powder particles forming the coating. The cracks in both deposits did not reach the substrates. They stopped in the coatings at distances of one-fourth or two-fifths from the substrate in the coatings cold sprayed on the Al alloy and steel, respectively, and started running in the direction perpendicular to the original one. The crack running through the deposit on steel was significantly smaller and less deep and wide than that on the Al alloy, and had a smaller extent (Figure 12b). It caused less system damage, which confirmed the higher force value needed to cause a loss of system stability.

**Figure 13:**
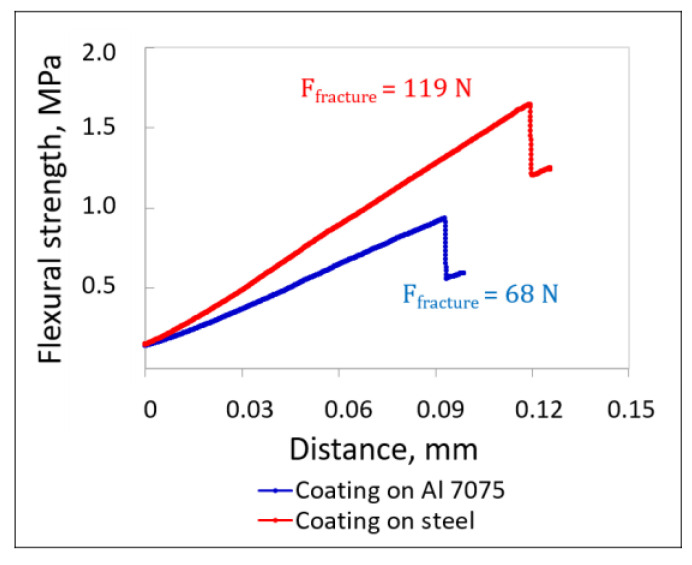
Flexural strength versus displacement of the pin during the three-point bending test of the substrate–coating systems.

The coating sprayed onto the steel substrate showed a flexural strength about 76% higher (1.60 MPa) than that sprayed onto the Al alloy (0.91 MPa), Figure 13. This proves that the coating deposited on steel substrates showed enhanced resistance to crack propagation. Both the aluminium alloy and steel substrates remained undamaged. Similar substrate effects were obtained for cermet (Cr_3_C_2_-25(Ni20Cr))–(Ni25C) deposits, with the observed cracks running up to the coating–substrate interface and then spreading to a limited extent parallel to the substrate surface [45]. In turn, Giu et al. [52] showed that the extent of interfacial cracking in bent samples tested during tensile stress depended on the coating thickness. More extensive interfacial cracking was observed in thicker coatings, showing that they were more likely to detach from the steel substrate [52].

**Figure 14:**
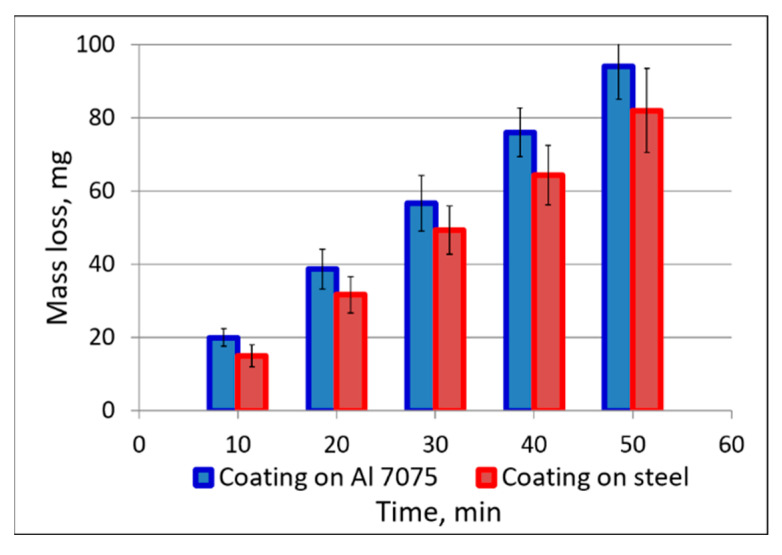
Mass loss versus time in the abrasive wear test.

The mass loss measurements using a T-07 tester after every 10 min of the abrasive wear tests with Al_2_O_3_ particles revealed that the substrate affects the abrasive properties of the cold sprayed coatings. After five cycles, the mass loss of the coating sprayed on the Al 7075 substrate was 94 mg, while that deposited on the steel substrate was 82 mg. As presented in Figure 14, the coatings on steel revealed about 20% lower wear than those on the Al alloy after each 10 min of the test. The better resistance to wear abrasion may suggest that this coating was more compact and formed from better-adhering powder particles. The coating’s lower mass loss corresponds to its higher microhardness (Figure 11) and more compact structure. Identical tests performed on cermet (Cr_3_C_2_-25(Ni20Cr))–(Ni25C) coatings showed a similar relationship, which indicates that cold sprayed coatings on a steel substrate are characterized by lower abrasive wear than those deposited on Al7075 alloy [45]. This is important when designing components with enhanced anti-wear properties.

**Figure 15:**
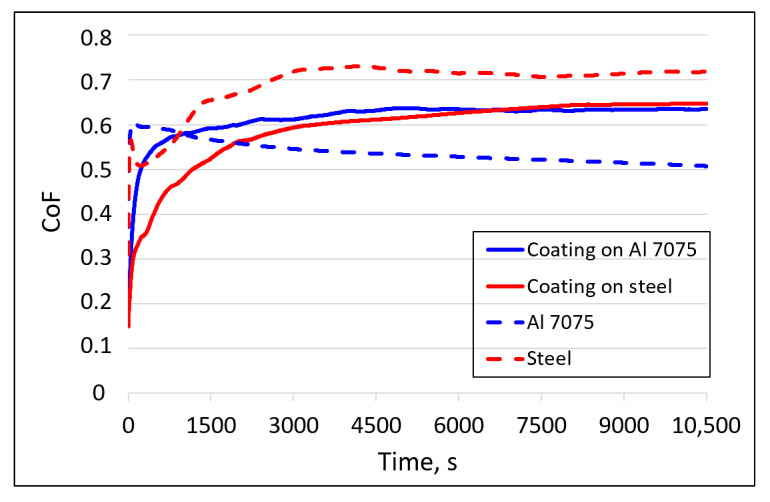
Friction coefficient changes with the measurement time determined under two loads for the coatings deposited on different substrates.

The coefficient of friction (CoF) and the wear index (W_v_) of coatings sprayed onto different substrates were determined based on the analysis of the coatings’ wear paths during operation in a sliding association with an Si_3_N_4_ ball in reciprocating motion. Figure 15 shows the changes in the CoF during the measurement determined for metallic glass coatings cold sprayed on the Al 7075 alloy and steel substrates and the friction coefficients for the substrates. The CoF curves for the coatings can be divided into two ranges. The first range covers the time from the beginning to about 1500 s, where the CoF increases significantly. This is due to the initial roughness changes into the so-called “working roughness”. The second part covers the time from 1500 s to the end of the measurement. During this time, complete stabilization did not occur, but the CoF had a higher value and a more stable trend. The CoF determined for the substrates stabilized only after about 3000 s. Figure 15 shows that the CoF for both coatings is the same (within the error limits), about 0.64. It is lower than for the steel substrate (about 0.72) and significantly higher than for the Al 7075 alloy (about 0.53). Although the CoF determined for the sliding association of the coating deposited on an Al alloy substrate and an Si_3_N_4_ ball is higher than that for the combination of the Al 7075 substrate and an Si_3_N_4_ ball, the wear index of the coating is incomparably lower than that for the Al 7075 alloy (Table 3). The CoF of these deposits was comparable to a cold sprayed Fe48Cr15Mo14C15B6Y2 amorphous coating measured under the same load (5 N) during 1200 s (about 0.55) [18]. Similar values of CoF (0.62, 0.68, and 0.66) were obtained by Yang et al. [53] for plasma-sprayed metallic glass coatings Fe43Cr16Mo16C5B20, Fe43Cr16Mo16C15B10, and (Fe0.43C0.16Mo0.16C0.15B0.10)98.5Y1.5, respectively. They were determined during wear tests with an Si_3_N_4_ counter sample of 4 mm in size and a load of 3 N. Slightly higher CoF values (0.7–0.85) were obtained by Milanti et al. [54] for partially amorphous HVOF-sprayed Fe-based coatings under 5 N with an alumina ball. The CoFs determined for Kuamet coatings were also lower than for plasma sprayed amorphous Fe72W10Cr4Ni3Mo2B4Si4C1 coatings sliding against an Si_3_N_4_ ball at a load of 6 N, which were in the range of 0.8–0.9 [55].

**Figure 16:**
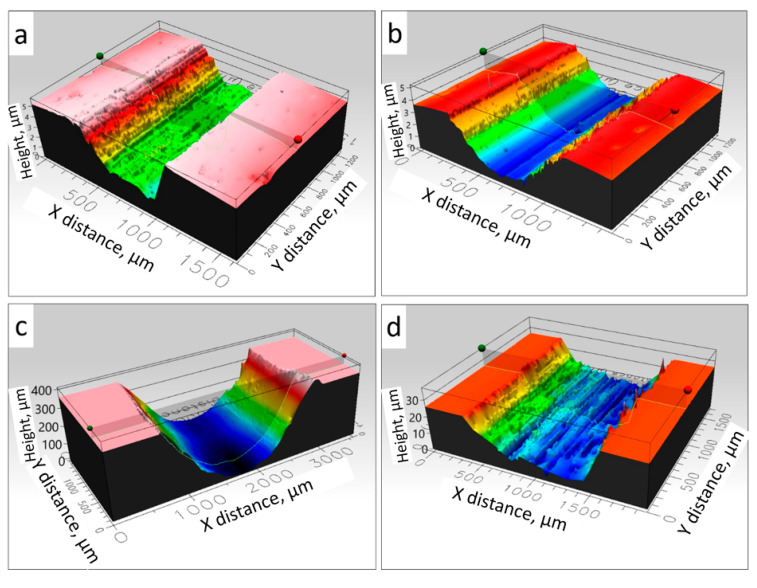
Examples of wear paths of coatings sprayed on the Al 7075 substrate (**a**) and steel substrate (**b**) and Al alloy substrate (**c**) and steel substrate (**d**).

As with CoF, the type of substrate on which the coating was sprayed did not show a significant effect on the deposit wear index. Both coatings had a similar wear index (W_v_) of 17.6·10^−6^ mm^3^/N·m and 17.2·10^−6^ mm^3^/N·m for the coating deposited on the Al7075 alloy and steel substrates, respectively, as shown in Table 3. It is worth emphasizing that the wear index of the examined coatings was much lower, almost by an order of magnitude, than that determined by Su et al. [18] for the cold sprayed Fe48Cr15Mo14C15B6Y2 amorphous deposits (2.1·10^−4^ mm^3^/N·m) and HVAF sprayed Fe48Cr15Mo14C15B6Y2 + Al_2_O_3_ (2.32·10^−4^ mm^3^/N·m) coatings during sliding wear tests with a zirconia counterpart. They were comparable to the wear rate of 1.9·10^−5^ mm^3^/N·m for partially amorphous deposits determined by Milanti et al. [54] in the ball-on-disk test. Also, An et al. [55] determined a similar wear rate (14–23·10^−6^ mm^3^/N·m) for the plasma sprayed Fe-based metallic glasses. For comparison purposes, test results for the substrate materials are also included. The wear indices of the substrates are significantly higher than those of the amorphous coatings cold sprayed on them. Hence, MG cold sprayed coatings from Kuamet 6B2 powder can be successfully used as wear-resistant coatings for aluminium alloy and steel elements. In turn, Cao et al. [40] showed that cold sprayed Fe25Cr20Mo1Si amorphous coatings reveal approximately three times lower weight loss than the 40Cr substrate during the wear test, where the friction pair coating–SiC ball was reciprocated. The W_v_ results are reflected in the wear paths shown in Figure 16. Both coatings were characterized by a similar wear path. The substrate wear paths are significantly more profound than the deposits, showing that the wear resistance of the Al alloy and steel substrates is worse than that of the amorphous alloy coatings. The deepest abrasions in the paths were observed for the Al alloy substrate, which confirms its highest wear.

**Figure 17:**
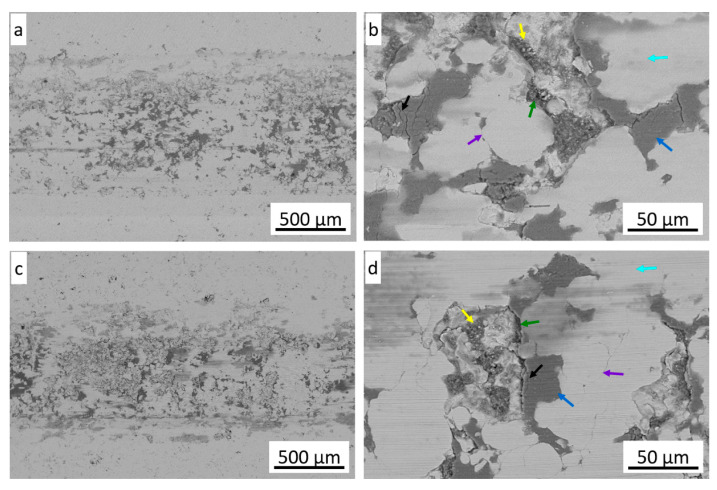
The SEM-BSE wear paths of the coating deposited on the Al alloy (**a**,**b**) and steel (**c**,**d**) substrate (blue—tribo layer, yellow—loose wear debris, green—groove, aqua—glaze layer, purple—grains of amorphous powder particles, black—cracks).

Figure 17 shows the morphology of the wear paths of the cold sprayed coatings observed by SEM. The paths look very similar for both coatings, have a similar width and show adhesive wear. They mainly reveal the plastic deformation with some debris and no visible coating fracture. In the scratch areas, one can distinguish the tribofilm layers resulting from the ball-coating friction pair with small cracks, loose wear products, grooves, and grain boundaries of amorphous particles.

## 4. Conclusions

This paper focuses on the differences in the mechanical properties and wear resistance of the Fe-based metallic glass coatings cold sprayed on different substrates by examining their microstructure, porosity, hardness, flexural strength, and wear behaviour. The identified microstructure features correlated with the examined properties will be a valuable input in knowledge to help predict the behaviour of coatings during practical applications. The most important conclusions resulting from the research are presented below.

1. Cold spraying of amorphous powders creates possibilities for forming thick (above 700 µm) metallic glass coatings, significantly thicker than those obtained in other thermal spray processes, exhibiting high mechanical properties and wear resistance.
2. The coatings cold sprayed on the Al 7075 alloy and steel substrates were characterized by a homogeneous and compact microstructure.
3. The coating–substrate systems observed after the three-point bending showed cracks appearing on the coating surfaces, which propagated in the coatings in the direction of the applied force. The flexural strength of the coating–steel substrate system was about 76% higher than in the case of the coating sprayed on the Al alloy.
4. The coating deposited on the steel substrate showed higher microhardness, better resistance to loose abrasive wear, and a slightly lower wear index when tested in the coating–Si_3_N_4_ ball tribological association.
5. Both coatings were characterized by a comparable CoF. This indicates that the type of the substrate (steel, Al 7075 alloy) had no effect on CoF.
6. Amorphous cold sprayed coatings can be successfully used to protect Al alloy and steel elements operating in dry friction conditions, as indicated by the low values of the wear index. The coatings exhibit similar wear and CoF characteristics; therefore, those cold sprayed on an aluminium alloy should be used where low component weight is a significant factor, while those deposited on steel can operate under bending conditions and at higher temperatures.

## Figures and Tables

**Table 1:** The chemical composition of the substrates [45].

Chemical Composition wt.%									
Al 7075 (EN 573-3:2019(E)) [46]									
Si	Fe	Cu	Mn	Mg	Cr	Zn	Ti	Others (max)	Al
0.4	0.5	1.2–2.0	0.3	2.1–2.9	0.18–0.28	5.1–6.1	0.2	0.2	Balance
X6CrNiTi18-10 steel (EN 10088-3:2014(E)) [47]									
C	Si	Mn	P	S	Cr	Ni	Others (max)		Fe
0.18	1.0	2.0	0.045	0.03	17–19	9–12	Ti:5xC-70		Balance

**Table 2:** The cold spraying parameters.

Parameters	Values
Working gas	50% N_2_ + 50% He
Gas pressure, MPa	3.5
Temperature, °C	700
Powder feeder rate, g/s	1.03
Standoff distance, mm	20
Speed of robot arm, mm/s	200
Number of passes	5

**Table 3:** Wear index determined for the coatings and substrate materials.

	Coating on Al 7075	Coating on the Steel	Al 7075 Alloy	X6CrNiTi18-10 Steel
**Wear index,** W_v_·10^−6^ [mm^3^/Nm]	17.6 ± 0.2	17.2 ± 1.2	2989 ± 135	173 ± 10

## Data Availability

The original contributions presented in this study are included in the article. Further inquiries can be directed to the corresponding author.
